# A Pragmatic Grouping Model for Bone-Only De Novo Metastatic Breast Cancer (MetS Protocol MF22-03) †

**DOI:** 10.3390/cancers17122033

**Published:** 2025-06-18

**Authors:** Berk Goktepe, Berkay Demirors, Kazim Senol, Serdar Ozbas, Efe Sezgin, Anthony Lucci, Atilla Soran

**Affiliations:** 1 Department of Surgery, Ege University, Izmir 35100, Türkiye; berkgoktepe@gmail.com; 2 Department of General Surgery, Bursa Yuksek Ihtisas Training and Research Hospital, Bursa 16140, Türkiye; drberkaydemirors@gmail.com; 3 Department of General Surgery, Uludag University, Bursa 16059, Türkiye; kazimsenol@gmail.com; 4 Private Practice, Ankara 06680, Türkiye; sozbas@yahoo.com; 5 Department of Food Engineering, Izmir Institute of Technology, Izmir 35433, Türkiye; efeszgn0@gmail.com; 6 Division of Breast Surgical Oncology, Department of Surgery, University of Texas MD Anderson Cancer Center, Houston, TX 77030, USA; alucci@mdanderson.org; 7 Division of Breast Surgical Oncology, Department of Surgery, University of Pittsburgh School of Medicine, Pittsburgh, PA 15213, USA

**Keywords:** stage IV breast cancer, novel staging system, multimodal therapy, primary tumor surgery, bone-only metastases, locoregional treatment, de novo metastatic breast cancer

## Abstract

De novo metastatic breast cancer (dnMBC) accounts for 3–10% of newly diagnosed cases, with 20–40% presenting as a bone-only metastatic disease, which can achieve survival outcomes exceeding 10 years with multimodal therapy. However, the role of multimodal therapy remains controversial in the guidelines. **Objective:** This study aims to identify dnBOMBC subgroups to develop a pragmatic staging system for guiding locoregional therapy decisions. **Materials and Methods:** Data from the MF07-01 phase III randomized trial (2021, median follow-up time (mFT): 40 months (range 1–131)) and the BOMET prospective multi-institutional registry trial (2021, mFT: 34 months (range 25–45)) were combined for analysis, including only patients who presented with bone-only metastases. Exclusion criteria were patients under 18 and those with a history of prior cancer or cancer metastases. Patients with missing data and positive surgical margins were excluded. Out of 770 patients, 589 were included. Survival analyses were first conducted according to molecular subgroups, after which patients were further stratified by hormone receptor status, human epidermal human epidermal growth factor receptor 2 (HER2) status, tumor grade, and clinical T (cT) stage. Group A (GrA) included hormone receptor (HR)-positive, low- or intermediate-grade tumors at any cT; HR-positive, high-grade tumors with cT0–3; or any HER2-positive tumors. Group B (GrB) included HR-positive, high-grade tumors with cT4 disease or any triple-negative (TN) tumors. **Results:** The hazard of death (HoD) was 43% lower in GrA than in GrB. Median OS was 65 months (39–104) for GrA patients and 44 months (28–72) for GrB patients (HR 0.57, 95% CI 0.41–0.78, *p* = 0.0003). Primary tumor surgery (PTS) significantly improved OS in GrA patients, regardless of the number of metastases (solitary: HR, 0.375, 95% CI 0.259–0.543, *p* < 0.001; multiple: HR 0.435, 95% CI 0.334–0.615, *p* < 0.001). Conversely, GrB patients did not experience a significant benefit from PTS. **Conclusions:** This study demonstrates that GrA patients have better OS than GrB patients, and PTS reduces the HoD in GrA patients compared to systemic therapy alone. These findings support using a modified staging system in dnBOBMC to identify patients who may benefit from multimodal therapy including PTS.

## Simple Summary

A refined staging model incorporating biological and anatomical tumor characteristics can better identify de novo bone-only metastatic breast cancer (dnBOMBC) patients who benefit from multimodal therapy, potentially guiding future treatment guidelines. A subgroup of dnBOMBC patients demonstrating overall survival (OS) comparable to Stage III breast cancer may be reclassified as Stage III D and thus directed toward locoregional therapy (LRT). Our study offers a practical model to identify those benefiting most from LRT, providing a more applicable framework than current nomograms.

## 1. Introduction

The current standard approach to the treatment of dnMBC is systemic therapy (ST); this is widely accepted in clinical practice. Contemporary guidelines continue to recommend ST as the primary modality. Local and regional treatments are typically used in select cases for palliative purposes, such as symptom control or the prevention of complications. However, dnMBC represents a highly heterogeneous disease group, with distinct subgroups exhibiting different prognoses. dnMBC re approximately 3–10% of new breast cancers (BCs), with bone being the most common metastatic site. In a study using the Surveillance, Epidemiology, and End Results (SEER) database including 18,322 patients, Wang et al. demonstrated that patients with bone-only metastases had a more favorable prognosis. Estrogen receptor (ER), progesterone receptor (PR), HER2, tumor grade, and genomic assays, along with tumor size, nodal status, and other disease-related factors such as the site and number of metastases, have all been shown to be associated with survival duration in patients with metastatic breast cancer (MBC) [1,2,3,4]. With multimodal therapy, these patients often achieve relatively more prolonged survival, with some studies showing an excess of 10 years [5,6]. Despite longer OS in dnBOMBC, current guidelines do not indicate which subgroups benefit most from ST and LRT, such as PTS and radiotherapy (RT). Traditional staging classifies all metastatic BCs as Stage IV, obscuring the substantial heterogeneity in prognosis for patients with different biological tumor subtypes and metastatic distributions. This highlights the need for a refined or “pragmatic” staging system for dnBOMBC.

In other malignancies like colon, lung, ovarian, and thyroid cancers, the American Joint Committee on Cancer (AJCC) has introduced metastatic subcategories to enhance prognostic accuracy [7,8,9,10]. Tamirisa et al. showed that reclassifying BC patients with supraclavicular metastases (cN3c) from Stage IV to IIIc improved survival, with multimodal therapy achieving a 5-year OS of 59%, compared to 28% in other groups [11]. Similarly, Plichta et al. [12] compared the AJCC seventh and eighth editions and found that 36.6% of patients were restaged, with 29.7% down-staged, including 94.1% of Stage IB and 82.5% of Stage IIIC cases. These findings highlight the potential impact of refined staging systems in improving outcomes for dnMBC patients [12].

A new staging system for dnMBC was recently proposed, along with an online calculator, using data from the National Cancer Database (NCDB) and Surveillance, Epidemiology, and End Results (SEER). This proposed staging integrates clinical and pathological variables, including T category, tumor grade, ER/PR/HER2 status, histology, metastatic sites (bone only vs. others), and the number of affected organ systems. Patients were stratified into Stages IV A–D based on 3-year OS rates (>70%, 50–70%, 25–50%, and <25%), with bone-only metastases identified as a favorable subgroup. While this model demonstrated the importance of stratifying dnMBC patients, the AJCC system still lacks classifications specific to dnMBC [13,14,15]. In this study, we aim to introduce subgroups for dnBOMBC patients based on immunohistochemical and anatomical features and test the hypothesis that some may be appropriately classified as Stage IIID.

## 2. Materials and Methods

This study is a subgroup data analysis from the MF07-01 phase III randomized and the BOMET prospective multi-institutional registry trials [5,16]. The MF07-01 trial randomly compares two standards of care in de novo Stage IV breast cancer. Randomization of this study launched in 2007 in Türkiye, and the Central Ethics Committee accepted the study at the time, with oral consent being the only condition. This is the main revision to the Clinical Trials Regulation that has been in force in Türkiye since 2014, after this study closed. As per the Health Insurance Portability and Accountability Act (HIPAA) regulations, registry trials such as the BOMET Registry trial are not required to obtain informed consent forms. The current study included only patients with bone metastases. Exclusion criteria were patients under 18 and those with a history of prior cancer or cancer metastases. Patients with missing clinical T or N stages, tumor grade, HR status, HER2 status, or metastatic disease site were excluded. All patients who underwent surgery had negative surgical margins. All patients who received LRT also received ST [17].

### 2.1. Patient Classification and Risk Grouping

Patients were initially categorized into four primary molecular groups based on HR and HER2 status. HR positivity was defined as the presence of either ER or PR expression. HER2 status was defined as negative (HER2(−)) in cases with immunohistochemistry (IHC) scores of 0 or 1+, and positive (HER2(+)) in cases with IHC 2+ and a confirmed FISH amplification, or directly for IHC 3+.

Among these groups, patients with HR(+)/HER2(−) tumors (Group 1) were further stratified into three subgroups according to tumor grade and cT stage:

- Group 1a: HR(+)/HER2(−), Grade 1–2, any cT stage.
- Group 1b: HR(+)/HER2(−), Grade 3, cT0–3.
- Group 1c: HR(+)/HER2(−), Grade 3, cT4.

The remaining molecular subgroups were defined as follows:

- Group 2: HR(−)/HER2(+).
- Group 3: HR(+)/HER2(+).
- Group 4: HR(−)/HER2(−) (TN).

For the purpose of risk stratification, patients in Groups 1a, 1b, 2, and 3 were collectively designated as Group A, representing cases with more favorable biological features and/or expected responsiveness to LRT. In contrast, patients in Group 1c and Group 4 were classified as Group B, which included tumors with either more aggressive local characteristics (e.g., Grade 3 and cT4 in Group 1c) or TN profiles with limited targeted treatment options.

This classification framework was developed to explore the prognostic impact of LRT across biologically distinct subgroups of dnBOMBC. The grouping system applied in this study was developed based on consistent findings from both our own prior analyses of the MF07-01 and MF14-01 BOMET datasets and supported by recent large-scale prognostic modeling studies in the literature [16,18]. These studies have repeatedly found HR status, HER2 status, tumor grade, and cT stage as the primary biological and anatomical factors associated with prognosis in dnMBC. Therefore, the present classification framework was grounded in these variables, aiming to reflect biologically meaningful differences in survival and responsiveness to locoregional therapy. This concept is supported by several recent publications that proposed prognostic scoring systems and nomograms incorporating these same core indicators [13,14,15,19].

The multimodal therapy in this study incorporated PTS, RT, and ST, providing a comprehensive approach to patient management. The OS of patients who received LRT in addition to ST was compared to those who received ST alone.

### 2.2. Statistical Analysis

Student *t*-tests were used to compare continuous variables with normal distribution between the groups. Violations of normal distribution were tested using the Shapiro–Wilk test, and the Wilcoxon rank-sum test was used for variables without normal distribution. Chi-square tests were used to compare the distribution of categorical variables. Survival outcomes were assessed using Kaplan–Meier curves and compared using the log-rank test. Hazard ratios (HRs) and corresponding 95% confidence intervals (CIs) were estimated using Cox proportional hazards regression models. Both univariate and multivariable Cox analyses were performed. Multivariable models were adjusted for relevant clinical and pathological covariates, including age at diagnosis, tumor subtype, histologic grade, clinical tumor stage, and use of systemic, locoregional therapies. *p*-values of less than 0.05 were considered statistically significant. Statistical analyses were conducted with R version 3.6.1 (R Foundation for Statistical Computing, Vienna, Austria, https://www.r-project.org) software packages and IBM SPSS Statistics (IBM Corp., Armonk, NY, ABD, USA) for Windows, version 22.0.

## 3. Results

**Figure 1:**
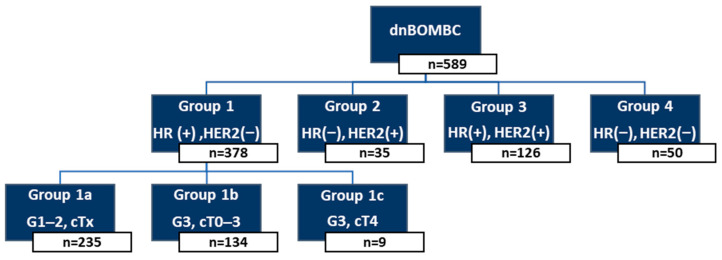
Classification according to the characteristics of the cases. dnBOMBC, de novo bone-only metastatic breast cancer; HR, hormone receptor; HER2, human epidermal growth factor receptor 2; G, grade.

This study included 589 patients with an average age of 53 years (range: 19–90). No statistically significant survival difference was observed among patients in Groups 1a, 1b, 2, and 3, with a median OS of 64, 72, 63, and 68 months, respectively (*p* = 0.67), so they were classified as Group A. In contrast, the median OS for patients in Group 1c and Group 4 was 41 months (range: 14.7–67.3) and 48 months (range 32.1–63.9), with no significant difference (*p* = 0.34), forming Group B (Figure 1).

Of the 589 patients in the study, 530 (89%) were in Group A and 59 (10%) were in Group B. The median follow-up was 55 months (range: 37–71.5). PTS was performed in 315 patients (53.48%), including 54.34% of patients in Group A and 45.76% in Group B.

**Figure 2:**
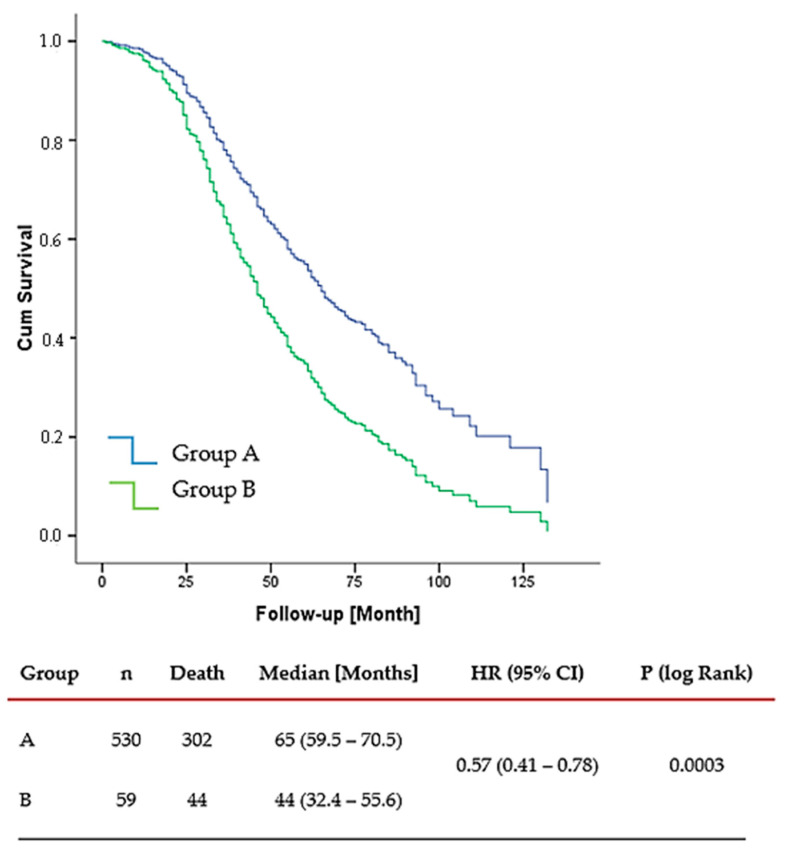
Overall Survival Group A vs. Group B. Overall survival was significantly longer in Group A compared to Group B.

OS was significantly longer in Group A compared to Group B, with a median OS of 65 months (range: 39–104) versus 44 months (range: 28–72), respectively. This stands for a 43% reduction in the hazard of death for Group A (HR 0.57, 95% CI: 0.41–0.78, *p* = 0.0003), indicating a meaningful survival advantage associated with more favorable tumor biology (Figure 2).

**Figure 3:**
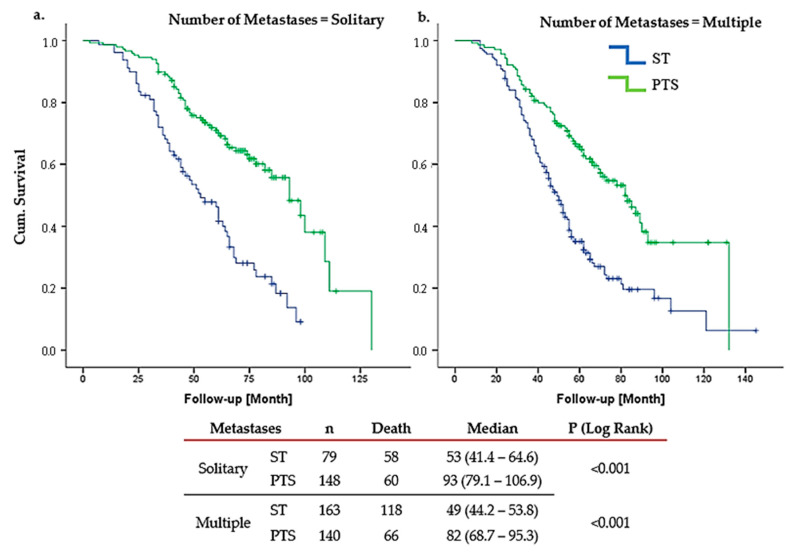
Group A overall survival PTS vs. ST (**a**). solitary metastases. (**b**). multiple metastases. PTS: primary tumor surgery, ST: sytemic therapy. In Group A, PTS significantly improved overall survival in both solitary and multiple metastatic cases.

LRT significantly improved OS in Group A. Among patients with solitary bone metastases, the median OS was 93 months (95% CI: 79.14–106.86) in the LRT group, compared to 53 months (95% CI: 41.44–64.56) in the ST-only group. This corresponds to an HR of 0.375, showing a 62.5% reduction in the risk of death for patients receiving LRT. The narrow confidence interval and highly significant *p*-value (*p* < 0.001) further support the reliability and clinical relevance of this finding. Similarly, in the subgroup of Group A patients with multiple bone metastases, those who received LRT demonstrated a notably prolonged survival, with a median OS of 82 months (95% CI: 68.74–95.26), compared to 49 months in the ST-only group. The observed HR of 0.453 reflects a substantial survival advantage associated with LRT, and the statistically significant *p*-value (*p* < 0.001) reinforces the consistency of this benefit across different metastatic burdens (Figure 3).

**Figure 4:**
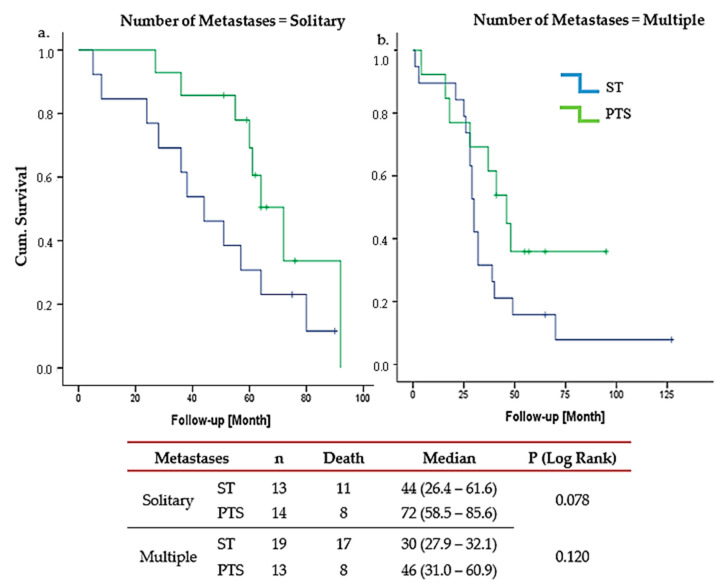
Group B overall survival PTS vs. ST. (**a**). solitary metastases; (**b**). multiple metastases. PTS: primary tumor surgery, ST: Sytemic therapy. Group B patients showed no OS benefit from PTS, regardless of bone metastasis count.

Conversely, Group B patients showed no OS benefit from LRT, regardless of bone metastasis number (solitary metastasis *p* = 0.07; multiple metastasis *p* = 0.12) (Figure 4). In Group 4, LRT is marginally significant only in solitary bone metastases (*p* = 0.04).

## 4. Discussion

dnMBC is defined as the presence of metastasis at initial BC diagnosis. The dnMBC rate among all MBC patients has risen from 20% to 30–40% [20]. The AJCC eighth edition classifies MBC as a single stage. Notably, nearly half of these patients present with oligometastatic disease, benefiting from a multimodal treatment, including ST, PTS, and RT. However, evidence is largely based on non-randomized studies. Careful selection of dnMBC patients is crucial to avoid overtreatment.

The term oligometastatic disease was first introduced in 1995, referring to patients with limited metastases and a better prognosis [21]. Although the definition remains debated, the most commonly accepted criterion is the presence of five or fewer metastatic lesions, regardless of the number of involved organs [22]. Oligometastatic disease is also characterized by the feasibility of definitive treatment for all metastatic sites [23]. Bone (41.1%) is the most frequent site, followed by the lung (22.4%), liver (7.3%), and brain (7.3%) [24].

Bone metastases play a crucial role in prognosis and treatment. The phase III MF07-01 trial found that after 10 years, 19% (95% CI: 13–28%) of patients undergoing surgery were alive versus 5% (95% CI: 2–12%) receiving ST alone (*p* < 0.0003), with the most significant benefit in ER/PR+, HER2+, and solitary bone metastases [5]. In patients with solitary bone metastases, LRT showed a 14-month median survival benefit more than ST only, with an HR of 0.55 (95% CI 0.36–0.86, *p* = 0.009). LRT and ST combination decreased mortality by 29% at 10 years in HR-positive subgroups (HR 0.71, 95% CI 0.59–0.86, *p* = 0.0003). No survival benefit was seen in TN, visceral metastases or multiple bone metastases.

The BOMET MF14-01 study underscores the efficacy of multimodal treatment, showing 5-year OS of 33% for ST-only vs. 72% for LRT+ST (HR, 0.40; 95% CI: 0.30–0.54, *p* < 0.0001). In solitary metastases, the 5-year OS was 45% in the ST-only and 75% in the LRT (*p* = 0.0005), while oligometastatic patients demonstrated 42% and 72% in the ST-only and LRT groups, respectively (*p* = 0.002). In multi-metastatic disease, the 5-year OS rates were 31% in the ST-only group and 69% in the LRT (*p* < 0.0001). For patients with more than five metastases, the survival rates dropped to 14% and 49% in the ST-only and LRT groups, respectively (*p* = 0.005). Additionally, in HR-positive dnBOMBC, the HoD was reduced by 72% in the multimodal treatment group. However, no statistically significant difference in HoD was observed in TN patients receiving multimodal therapy compared to those treated with ST only (HR 0.51, 95% CI: 0.24–1.10) [16]. Similarly, our study demonstrated the survival benefits of LRT, particularly for Group A patients, who had a median OS of 65 months (range: 39–104) compared to 44 months (range: 28–72) in Group B (HR: 0.57; 95% CI: 0.41–0.78; *p* = 0.0003). Notably, among Group A patients with solitary bone metastases, the median OS in the LRT group was 93 months (95% CI: 79.14–106.86), compared to 53 months (95% CI: 41.44–64.56) in the ST-only group (HR: 0.375; 95% CI: 0.259–0.543; *p* < 0.001).

As previously mentioned, the BOMET study demonstrated that LRT had a positive impact on OS in patients with solitary, oligo, or multiple bone metastases, regardless of the number of lesions. Therefore, when designing our study, we defined Group A and Group B without considering the number of bone metastases. Furthermore, subgroup analyses evaluating the impact of metastatic burden on OS among patients who received LRT revealed no statistically significant association in either group.

Several retrospective studies confirm multimodal treatment benefits, particularly in younger patients, ≤3 metastases, and HR+/HER2− or HER2+ tumors. Thomas et al., using SEER data from 21,372 patients, found that surgery improved median OS in bone-only metastatic (BOM) patients (19 vs. 28 months; *p* < 0.001) and enhanced 10-year survival (OR 3.61, 95% CI 2.89–4.50). Similar trends were found in studies by Lane (53 vs. 38 months; *p* < 0.001), Pons-Tostivint (62 vs. 46 months; *p* < 0.001), Lopez-Tarruella (40 vs. 22 months; *p* < 0.0001), Cady (35 vs. 24 months; *p* = 0.021), and Shien (27 vs. 22 months, *p* = 0.049), confirming the impact of LRT, especially in limited bone metastases. Xiong et al. reported marked OS benefits in patients with ≤3 metastases (78 vs. 37 months; *p* = 0.002), while Kwong and Co observed improved 5-year OS rates with surgery after ST (43.9% vs. 33.9%; *p* = 0.0026) [25,26,27,28,29,30,31,32]. In our study, Group A patients benefited significantly from LRT regardless of the number of metastases. For solitary metastatic patients, the HR was 0.37 (95% CI 0.25–0.54; *p* < 0.001), while for patients with multiple metastases, the HR was 0.45 (95% CI 0.33–0.61; *p* < 0.001).

Conversely, the TATA and ECOG-ACRIN 2108 trials found no OS benefit from LRT. TATA trial reported improved locoregional progression-free survival (LPFS) but worse distant progression-free survival (PFS), while ECOG-ACRIN found no significant OS or PFS improvement. However, both had patient selection limitations [33,34]. The ECOG-ACRIN E2108 trial has faced criticism due to certain methodological limitations that may have influenced the interpretation of its results. Notably, negative surgical margins were not achieved in approximately 20% of patients assigned to the LRT group, which represents a significant limitation when evaluating the efficacy of surgical intervention. Cases with visceral metastases, which are known to have a poor prognosis and are less likely to benefit from LRT, were also included. Patients with bone-only metastases accounted for only 37.7% of the entire cohort. Furthermore, 14% of patients in the LRT arm did not undergo surgery despite allocation, and 15% did not receive RT following breast-conserving surgery, indicating issues with treatment adherence that may have impacted outcomes. A prospective trial by Abo-Touk showed higher 2-year OS (LRT 46% vs. ST-only 22%) but was not statistically significant (HR 0.346, 95% CI 0.031–3.817; *p* = 0.085). Although not statistically significant, they suggest LRT may be beneficial for patients based on bone metastases, the extent and location of metastases, and clinical T and N stages [35]. A Japanese trial at ASCO 2023 found LRT + ST improved LPFS (63 vs. 20 months, *p* < 0.0001), particularly in ER+, premenopausal, single-organ metastases, especially BOM [36]. Similarly, our study found that ER positivity, BOM, and single-organ metastases were common variables that positively affect survival, although disease-free survival data were unavailable.

In contrast, our study demonstrated clear survival advantages with LRT, particularly for Group A patients with solitary metastases, where the median OS was 93 months (95% CI: 79.14–106.86) compared to 53 months (95% CI: 41.44–64.56) in the ST-only group (HR 0.375, 95% CI: 0.259–0.543, *p* < 0.001). In Group B, LRT is marginally significant only in Group 4 (TN group) with solitary bone metastases (*p* = 0.04). Although the sample size is very small, this exploratory subgroup finding needs to be validated in a larger study focusing on TN patients. Almost all studies, including RCTs, have revealed no difference in OS among TN patients, while a few retrospective studies have shown the efficacy of LRT in patients with de novo MBC, including TN tumors and non-responders to ST [28]. In general, the real-world approach, as indicated in the MF07-10 RCT, is that unless we have a larger sample size, studies in this subset of patients (TN) that show an OS benefit with LRT should not be emphasized.

Meta-analyses also confirm LRT’s impact. Chongxi Ren et al. found no OS benefit (HR 0.87; 95% CI 0.68–1.11; *p* = 0.265) but notable LPFS improvement (HR 0.27, 95% CI 0.19–0.38, *p* < 0.001) [37]. Similarly, another meta-analysis found an improvement in LPFS, particularly in patients with BOM (HR 0.18; *p* = 0.017) [38]. Weikai Xiao et al. showed better distant PFS (HR 0.42, *p* < 0.001), particularly in solitary metastases, bone-only disease, and negative surgical margins [39].

Our study corroborates the survival advantages of LRT, especially in selected dnBOM patients, particularly Group A patients as the most likely to benefit from LRT and provides a pragmatic framework for refining patient selection and optimizing treatment outcomes.

Targeted therapies further improve dnMBC survival. The CLEOPATRA trial demonstrated pertuzumab + trastuzumab + docetaxel extended OS (HR 0.60, CI 0.50–0.72), while PALOMA-2 (27.9 vs. 22 months; HR 0.61) and MONALEESA-3 trial (59.9 vs. 50.9 months; HR 0.62) confirmed CDK 4/6 inhibitors’ efficacy. KEYNOTE-355 trial demonstrated pembrolizumab improved PFS in metastatic TNBC (7.6 vs. 5.6 months; HR 0.74, *p* = 0.0014) [40,41,42,43]. While our study did not include immunotherapy or CDK 4/6 inhibitors, our results align with findings in low tumor burden and HR+ patients benefiting from multimodality treatment.

There is an unmet need for research to evaluate the integration of new therapy regimens for enhanced survival for specific patient subgroups.

Patients with dnMBC are a diverse group in terms of prognosis and necessitating personalized treatment approaches [3,4,44]. In the study conducted by Wang et al., which included 18,322 cases, patients with HR(+), HER2(−), and bone-only metastases were shown to have the most favourable prognosis [45]. Similarly, the MF07-01 trial found that patients who were HR(+), HER2(−), under 55 years of age, and had solitary bone metastases derived the greatest LRT [5]. The prospective randomized phase III JCOG1017 trial also demonstrated in subgroup analyses that LRT improved survival in patients with ER-positive tumors, premenopausal status, or single-organ metastasis [36]. Taken together, these findings suggest the existence of a common subgroup among Stage IV patients who not only have a better prognosis but also benefit from LRT. The AJCC eighth edition staging system incorporates immunohistochemical characteristics and anatomical factors for BC staging but does not provide specific adjustments for dnMBC. New staging systems are crucial in more accurately assessing the prognosis of dnMBC patients, allowing for the more effective planning of ST and LRT. In response, several studies have proposed novel staging systems and nomograms to better predict outcomes in dnMBC patients. Lin et al. identified M1a (solitary bone metastases or a single non-liver/brain site) as experiencing the most significant benefit from LRT (HR 0.57; 95% CI 0.48–0.67) [46]. Wang et al. proposed a nomogram showing significantly longer OS in surgery vs. non-surgery groups (53 vs. 33 months; HR 0.64; *p* < 0.001). Factors affecting the benefit of LRT included histological grade, T stage, molecular subtype, and the location of metastases, with BOM identified as a strong positive factor [13]. Yoo et al. used a survival prediction model, where surgery showed significantly better median OS (53 vs. 31 months; *p* < 0.001), identifying younger, HR-positive, with smaller tumors, low metastatic burden, and BOM patients as ideal candidates for LRT, including PTS, RT, and metastatic resection [47]. Kommalapati et al.’s prognostic model demonstrated that BOM, ER positivity, and low tumor grade were associated with better OS, while high tumor burden and aggressive histology were negative factors [14]. Our study aligns with these findings, showing that LRT significantly improved OS in BOM patients, particularly solitary metastasis (93 vs. 53 months; *p* < 0.001) and multiple bone metastases (82 vs. 49 months; *p* < 0.001).

Plichta et al. (2023) [48] proposed a novel staging system for dnMBC based on metastatic site, ER and HER2 status, clinical T stage, and tumor grade. The categorized metastatic Stage IV for these stages was 73.2% for IVA, 61.9% for IVB, 40.1% for IVC, and 17% for IVD (*p* < 0.001). In the paper, the authors proposed a new staging system using a web-based calculator that can be used to restage such patients. This nomogram is for all dnMBC patients using data pulled from SEER and NCDB datasets. In their paper, S1–4 was defined as the number of organ systems involved, such that a patient with BOM would be assigned “S2” regardless of the number of bone metastases and liver metastases. Patients who have one organ metastasis and ER(+)/PR(+)/T0-3/HER2(−) and grade 1–2/ductal histology/BOM are staged as Stage IVB, and median 3-year OS was 58.4% and 64.2% in cohort samples named validation one and validation two, respectively [48]. In our proposed model of dnBOMBC data, this patient would fit into Group 1a. For solitary bone metastasis, the median OS was 93 months in the LRT group compared to 53 months in the ST-only group (*p* < 0.001), with a median follow-up of 55 months. For the same patient characteristics with multiple bone metastases, the median OS was 82 months in the LRT group—33 months longer than in the ST-only group (*p* < 0.001) in our proposed model. While restaging dnMBC, we think it would be more accurate to separate bone metastasis from non-bone metastasis. The nomogram developed by Kommalapati et al. employs a prognostic scoring system ranging from 0 to 17. Patients with scores between 0 and 7 are classified as Group 1, representing those with a more favorable prognosis, while those with scores from 8 to 17 are placed in Group 2, indicating a poorer prognosis [14]. However, this model does not aim to predict which patients with MBC will benefit from surgery. Rather, it is designed to estimate OS irrespective of specific treatment modalities. Similarly, widely accessible tools such as the MD Anderson Cancer Center Stage IV Metastatic Breast Cancer OS Calculator (https://biostatistics.mdanderson.org/BreastCancerSurvival) and the Duke Metastatic Breast Cancer Prognosis Calculator (https://mbc.ocsresearch.com/#/) also focus solely on predicting OS and do not offer guidance on the likely benefit of LRT. In contrast, our proposed pragmatic grouping model seeks to identify which subgroups of dnBOMBC patients may derive the greatest survival benefit from LRT, thereby providing clinically actionable insight beyond prognosis alone.

While existing models focus on histology and molecular subtypes, future refinements should incorporate additional factors such as genomics and circulating tumor cell/DNA burden [49,50]. Our analysis of the MF07-01 and MF14-01 BOMET studies indicated that isolated bone metastasis is a key prognostic factor, and thus, we focused our study on this patient group. Meta-analyses and prospective studies consistently identify HR status, HER2 status, tumor grade, and cT stage as prognostic factors [51]. We utilized a pragmatic algorithm similar to that proposed by Plichta et al. to define subgroups based on these prognostic factors. Our findings revealed that higher tumor grade and cT stage were associated with poorer outcomes in HR-positive patients, while TN was a significantly poor prognostic factor. Notably, LRT in Group B did not provide a survival benefit.

In our study, Group A OS was comparable to Stage IIIC, with a median OS of 62 months. Qiu et al. reported a median OS of approximately 54 months, while Ai et al. noted 76 months, with variations depending on molecular subtypes and clinical characteristics in Stage IIIC [52]. In our study at 5 years, the median OS for Group B was 44 months, while for Group A, this could not be determined due to a substantial proportion of patients still alive (95% CI: 32.4–55.6 months; *p* = 0.001). Ai et al. showed a 5-year OS of 61.7%, while Qiu et al. reported 51.72%, with variations observed across N3 subgroups in Stage III [52,53]. In our study at 10 years, OS declined to 18% overall, with 19% for Group A and 12% for Group B, and median OS was 65 months for Group A (95% CI: 59.5–70.5 months) and 44 months for Group B (95% CI: 32.4–55.6 months; *p* = 0.0003). Although limited 10-year OS data for Stage IIIC are available in the literature, indicating poor outcomes, particularly for N3c disease, our study shows similar OS compared to Stage IIIC [52,53].

This study has certain limitations. One key limitation is the combination of two distinct datasets: the MF07-01 study, a phase III prospective randomized trial, and the MF14-01 BOMET study, a prospective multi-institutional registry cohort. While all patients included from the MF14-01 BOMET cohort had bone-only metastases at the time of breast cancer diagnosis, only 46% of patients in the MF07-01 trial presented with bone-only disease at initial staging. Therefore, only patients with bone metastases from the MF07-01 study were included in the analysis. Furthermore, the inclusion of registry-based data introduces the potential for selection bias, as treatment allocation (surgery vs. no surgery) in the MF14-01 cohort was not randomized and may have been influenced by physician judgment or patient preference. However, we would like to emphasize that the survival benefit observed with LRT is not solely supported by retrospective or registry-based data. The recently published prospective, randomized, phase III JCOG1017 trial demonstrated in subgroup analyses that LRT improved survival in patients with ER-positive tumors, premenopausal status, or single-organ metastasis [36]. These findings not only strengthen the potential value of surgical intervention in appropriately selected patients but also indicate that similar subgroups benefit from LRT in prospective trials as well. The second limitation was that both studies did not require a metastatic site biopsy, but two different imaging modalities were used to confirm the bone metastases. A further limitation was that metastatic site intervention was left to the institution’s choice.

Over time, ST has evolved, and various modalities have been applied in different studies, including ours, which may influence outcomes. Although all patients received ST, the treatments were not uniform. As new drugs became part of standard care over the years, some patients received more advanced therapies, which may have affected their prognosis. Additionally, the limited survival rates of metastatic patients often result in relatively short follow-up periods in many studies. To more accurately identify the patient groups that will derive the greatest benefit, it is essential to conduct studies with longer follow-up durations. Therefore, increasing the number of studies with extended follow-up is crucial for refining treatment strategies and improving patient outcomes.

## 5. Conclusions

LRT is a standard part of multimodal therapy for Stage III BC, but its role in Stage IV BC remains controversial. When a subgroup of dnBOMBC patients demonstrates OS similar to Stage III BC and receives multimodal therapy, they might be reclassified as Stage III D and prioritized for LRT. Our study presents a practical grouping model that distinguishes patients most likely to benefit from LRT, providing a more applicable framework than current nomograms. Further large-scale prospective studies are essential to validate these findings and refine the proposed staging system, providing clear guidance for integrating LRT into dnBOMBC treatment.

## Data Availability

The data of the present study is not publicly available.

## Abbreviations

The following abbreviations are used in this manuscript:

OS	Overall survival
LRT	Locoregional therapy
dnMBC	De novo metastatic breast cancer
cT	clinical T
HER2	Human epidermal human Epidermal growth factor Receptor 2
mFT	Median follow-up time
GrA	Group A
HR	Hormone receptor
GrB	Group B
HoD	The hazard of death
PTS	Primary tumor surgery
ST	Systemic therapy
BC	Breast cancer
ER	Estrogen receptor
PR	Progesterone receptor
MBC	Metastatic breast cancer
RT	Radiotherapy
AJCC	American Joint Committee on Cancer
NCDB	National Cancer Database
SEER	Surveillance, Epidemiology, and End Results
IHC	Immunohistochemistry
CI	Confidence intervals
BOM	Bone-only metastatic
LPFS	Locoregional progression-free survival
PFS	Progression-free survival
TN	Triple-negative
